# Genetic analyses reveal demographic decline and population differentiation in an endangered social carnivore, Asiatic wild dog

**DOI:** 10.1038/s41598-021-95918-3

**Published:** 2021-08-12

**Authors:** Shrushti Modi, Samrat Mondol, Parag Nigam, Bilal Habib

**Affiliations:** Wildlife Institute of India, Chandrabani, Dehradun, 248001 India

**Keywords:** Ecology, Genetics

## Abstract

Deforestation and agricultural intensification have resulted in an alarming change in the global land cover over the past 300 years, posing a threat to species conservation. Dhole is a monophyletic, social canid and, being an endangered and highly forest-dependent species, is more prone to the loss of favorable habitat in the Anthropocene. We determined the genetic differentiation and demographic history of dhole across the tiger reserves of Maharashtra using the microsatellite data of 305 individuals. Simulation-based analyses revealed a 77–85% decline in the major dhole sub-populations. Protected areas have provided refuge to the historically declining dhole population resulting in clustering with strong genetic structure in the remnant dhole population. The historical population decline coincides with the extreme events in the landscape over the past 300 years. The study highlights the pattern of genetic differentiation and diversity of a highly forest-dependent species which can be associated with the loss of forest cover outside tiger reserves. It also warrants attention to develop conservation plans for the remnant surviving population of dholes in India.

## Introduction

On the backdrop of rampant global industrialisation, urbanisation, and agricultural intensification, long-term survival of most wild animals and their habitats are severely challenged by drastic reduction in available habitats through fragmentation^1^. In the most intensified form, fragmentation events create detrimental edge effects along the boundaries of habitat patches leading to restricted animal movement and gene flow, severed landscape connectivity and drastic retrogressive demographic changes in the long run^2^. Although species adapt differently to such pressures^3^, generally animals having large body size with apex position in the food chain^4^, low growth rates^5^, wide home ranges^6^, and habitat specialists^7^ are at higher risk of facing the detrimental effects of fragmentation. In this regard, the mammalian carnivore guild is one of the most fragmentation-effected groups of species, making them the ecological indicators of landscape connectivity^8^. Large number of studies demonstrated the impacts of habitat fragmentation on carnivores (for example, ocelot^9^; African wild dog^10^; mountain lion and coyote^11^; gray wolf^12^) and established that habitat-specialist pack-living carnivores are more prone to degraded habitats owing to their smaller niche breadth, smaller range of dispersal^8^ and allee effect^13^.

Asiatic wild dog (*Cuon alpinus*) or dhole is a typical example of a pack living, habitat-specialist, apex carnivore. They are considered ‘Endangered’ by IUCN under criteria C2a(i) (small, declining and fragmented population with less than 2500 mature individuals) and are already at serious risk from habitat loss, prey depletion, disease transmission from domestic dog, human persecution and interspecific competition^14^. Their global population is approximately 4,500–10,500 with only 949–2,215 mature individuals with a decreasing population trend^14^. The Indian subcontinent is the major stronghold for the remnant dhole populations^14^ distributed mostly within the forested areas of the Western Ghats and the central Indian landscape. Several smaller populations have been reported from north-eastern India, Eastern Ghats and the Himalayan region^15,16^, but their long-term viability is under serious concern due to low population sizes. Throughout its distribution in India, this obligate forest-dwelling species is greatly affected by habitat loss (60% loss of its historical range)^17^ mostly from agriculture intensification, urbanisation and developmental activities^14^, leading to continuous insularisation of these populations along with restricted dispersal events. These fragmented populations may suffer from a reduction in genetic diversity^18^ and genetic drift at a longer temporal scale, leading to possible strong population structure and inbreeding depression^19^. Despite the knowledge of these ongoing situations of dhole biology and conservation, appropriate studies to verify genetic diversity and differentiation patterns are limited^14^. To date, most of the studies have focused on their behaviour^20–22^, occupancy^23^, population pattern^24,25^, genetics^26–28^ at local/regional scales, still an in-depth understanding of population/demographic patterns are lacking.

In this paper, we investigated the patterns of genetic differentiation, diversity and demography in the dhole population across Maharashtra, a part of a larger landscape of Central India, having possibly the largest dhole population^29^. Using non-invasive genetic tools, we evaluated (1) the extent of dhole genetic diversity across all known dhole habitats in the state; (2) population structure of dhole in this area; and (3) demographic history of the major populations within Maharashtra. We addressed these questions using 12 microsatellite loci^26^ surveyed in 305 individual dholes from six protected areas. Finally, we interpret the results in the lights of dhole ecology and historical changes on dhole habitat in the Central Indian Landscape.

## Results

### Genetic diversity

A total of 623 samples were collected from six protected areas during the study period (2016–2019). We identified 590 dhole faeces through species-specific molecular assay^27^, attaining an amplification success of 94%. Using a panel of 12 microsatellite markers^26^, we generated a dataset of 349 genotypes attaining a success rate of 59.1%. Out of them, 305 were identified as unique genotypes while 44 genotypes were removed as replicates from further analysis. Out of these, 101 individual genotypes were used in a previous study^26^, while 204 individual genotypes from three protected areas (TATR, STR, NNTR) were identified in this study. These loci provided a cumulative misidentification rate or PID_(unbiased)_ and PID_(sibs)_ value of 1.09 × 10^−10^ and 1.06 × 10^−4^, respectively, indicating a statistically robust value for dhole individual identification. Overall, the panel showed a low genotyping error rate where the mean allelic dropout rate was 0.040 per allele per locus, mean false allele frequency was 0.071 per allele per locus, and null allele frequency was 0.01, respectively. The genotyping error rate is within the threshold of 20% suggested for non-invasive population level studies^30,31^. The panel showed no evidence for strong linkage disequilibrium between any pair of loci. However, two to four loci from the panel were out of HW equilibrium in individual population, but not a single loci was found to be out of HW equilibrium in all populations (Supplementary Table 1)^26^. Mean pairwise relatedness was low for all the sub-populations in the four estimators (Supplementary Table 3).

### Population structure

**Figure 1:**
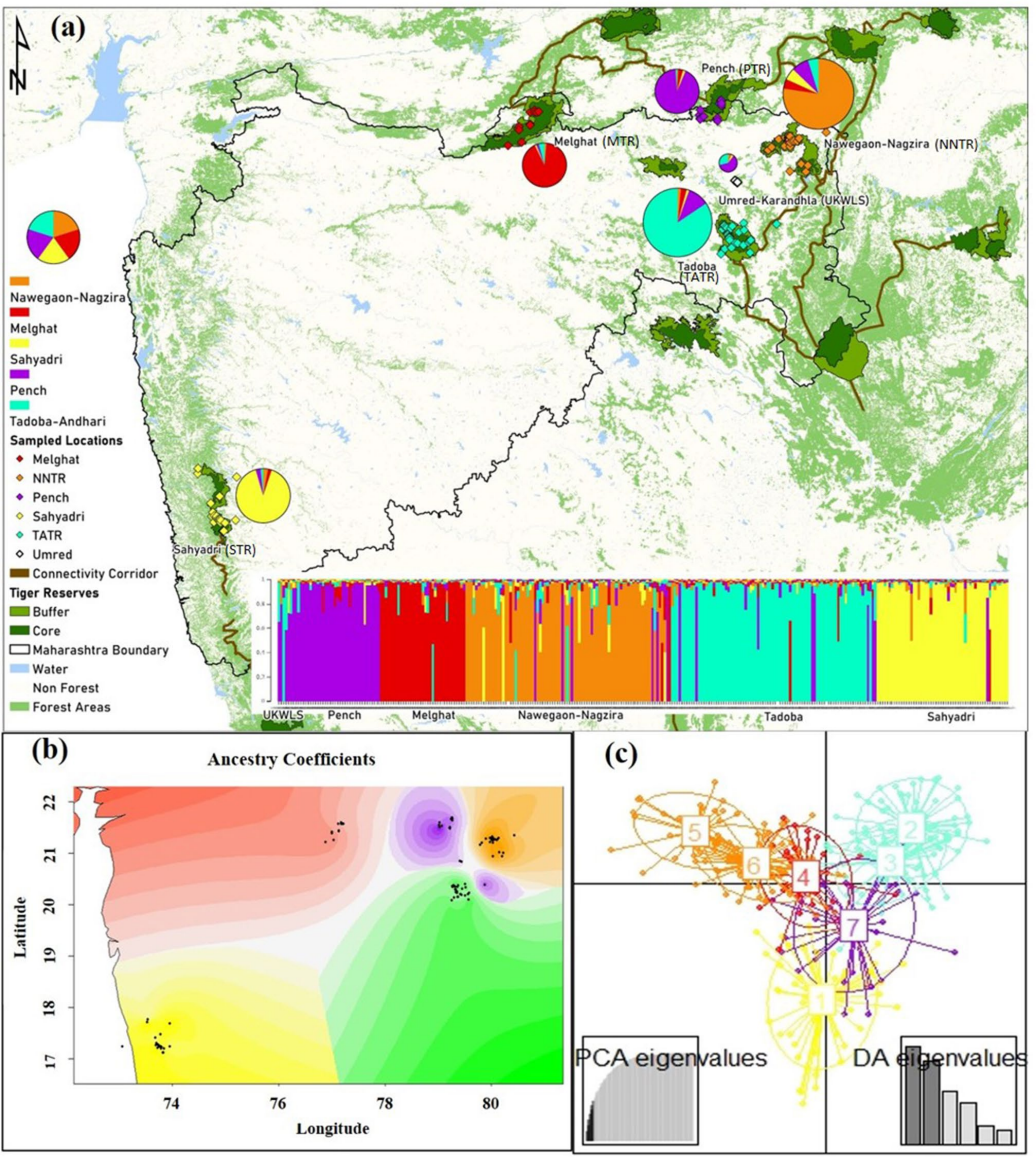
Dhole sampling and population structure across the study area. (**a**) shows the locations of the unique dhole individuals (n = 305) from the sampled protected areas (Nawegaon-Nagzira Tiger Reserve-NNTR, Melghat Tiger Reserve-MTR, Sahyadri Tiger Reserve-STR, Pench Tiger Reserve-PTR, Tadoba-Andhari Tiger Reserve-TATR, Umred Karandhla WLS-UKWLS) , along with population genetic structure of 305 dhole individual genotypes derived using a Bayesian clustering approach implemented in STRUCTURE. Each color represents a cluster and a single bar plot represents the individual. The-X axis represents the population while the Y-axis corresponds to the probability of assignment of an individual to each cluster. The pie chart presented for each protected area represents the respective proportion of genetic assignment in each population. The size of the pie-chart is proportional to the number of individual genotypes at each site. (**b**) represents the STRUCTURE ancestry coefficient (Q-matrix) through colour gradient for respective areas. (**c**) shows the genetic clusters (k = 7) from DAPC analysis. The TATR (cluster 2 and 3) and NNTR (cluster 5 and 6) populations show two overlapping clusters, making a total of five genetic subpopulations. Study area map was created using ArcGIS 10.3 (https://enterprise.arcgis.com/en/portal/10.3/use/get-started-with-maps.htm). (**c**) was generated using the package “adegenet” in R studio R Core Team (2019). R: A language and environment for statistical computing. R Foundation for Statistical Computing, Vienna, Austria. (https://www.R-project.org/).

Our sampling strategy focused on maximum coverage of unique individuals across a relatively small region of dhole distribution to assess any possible dhole population structure. Bayesian clustering analysis with 12 microsatellite loci showed five distinct genetic groups (K = 5 clusters) (Supplementary Fig. 1a,b) (Fig. 1a). The ancestry coefficient (Q-matrix) indicated five different focal ancestry points, as presented in Fig. 1b. Majority of the individuals (n = 285, 93.5%) showed group-specific ancestries, while a few individuals from UKWLS (n = 9), NNTR (n = 9), MTR (n = 5), TATR (n = 5), STR (n = 4) and PTR (n = 2) showed mixed ancestry signals. Careful investigation revealed five genetic clusters i.e. PTR (n = 33), MTR (n = 35), NNTR (n = 90), TATR (n = 84) and STR (n = 54). Structure plot for k = 2 to k = 4 also followed the same pattern (Supplementary Fig. 1d).

**Figure 2:**
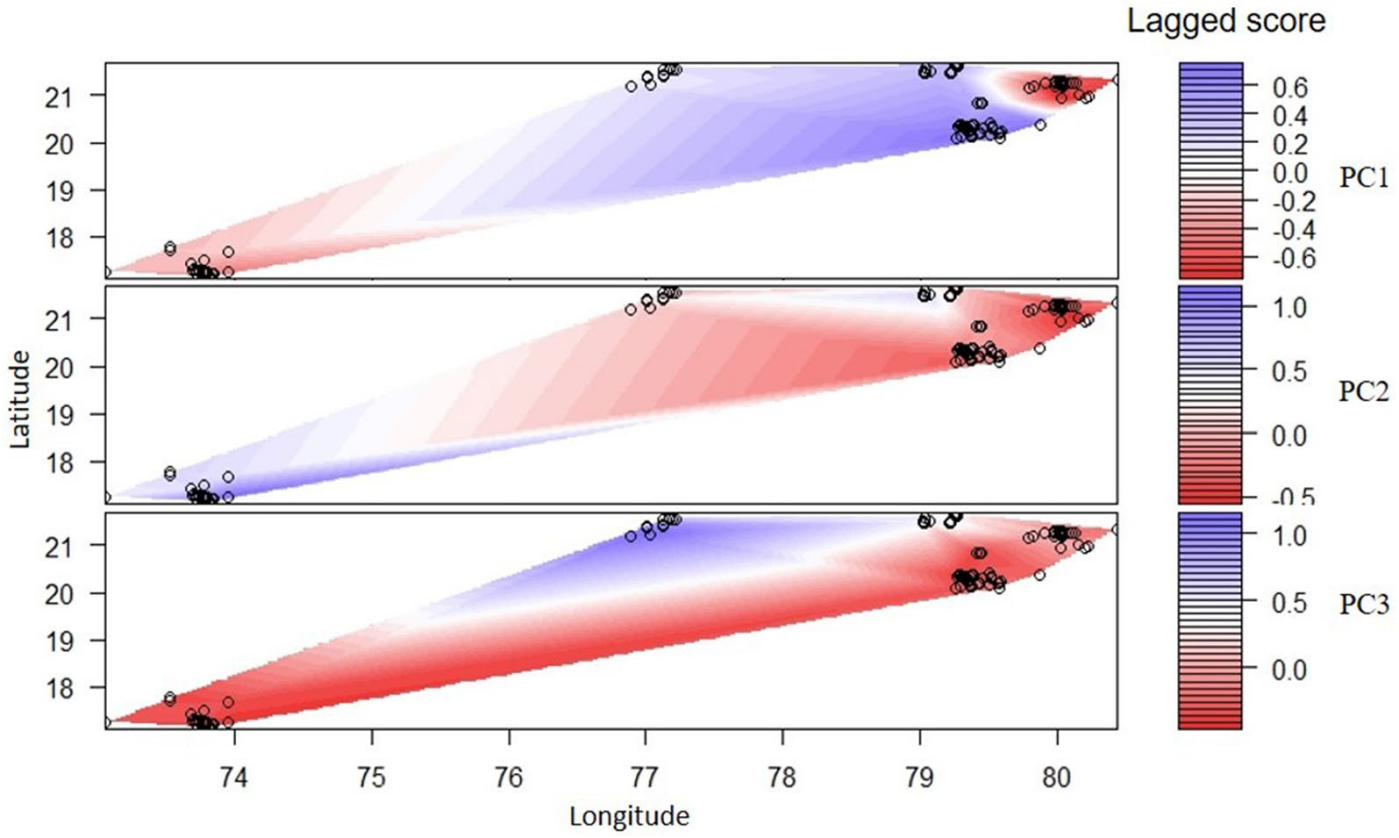
Results of genetic differentiation from sPCA analysis. The results are presented as heatmaps where higher differentiation corresponds to positive eigenvalue score of significant global genetic structure (presented as red colour), whereas lower values are shown as blue colour. The black circles represent the unique dhole individuals from different areas of this landscape. The figure was generated using the package “adegenet” in R studio R Core Team (2019). R: A language and environment for statistical computing. R Foundation for Statistical Computing, Vienna, Austria. (https://www.R-project.org/).

The BIC value from DAPC analyses suggested k = 7 with the lowest value, after which there is a subtle difference in the BIC value (Supplementary Fig. 1c). The seven identified clusters represent four clusters overlapping between TATR and NNTR, respectively (Fig. 1c). Subsequent DAPC runs with a prior value of K = 5 showed the same pattern seen in the STRUCTURE analysis. sPCA analysis revealed highly significant global (p = 0.0001) but non-significant (p = 0.4327) local spatial structures (Supplementary Fig. 2), indicating strong signatures of between population separations. Assessment of three major global principal component axes (based on eigenvalue) (Supplementary Fig. 3) indicates strong structure among STR, NNTR and TATR-MTR-PTR with PC1, STR and TATR-NNTR-MTR with PC2 and MTR with other subpopulations with PC3 (Fig. 2), corroborating with the earlier results. Combinedly, we interpret that our sampling area has five genetic clusters. Two independent analyses of population differentiation indices (G’st and Jost D) reveal significant levels of genetic differences among these five clusters. The G’st values ranged between 0.22–0.40, with the highest differentiation found between MTR-STR (0.40) and MTR-PTR (0.40), respectively, and the lowest value between PTR-UKWLS(0.20). Table 1 shows the cluster-wise genetic differentiation values for both indices.

**Table 1:** Pairwise value for G’st (lower diagonal) and Jost’s D (upper diagonal).

G’st/D Jost	UKWLS	PTR	MTR	NNTR	TATR	STR
UKWLS		0.095(0.04–0.16)	0.229(0.16–0.32)	0.146(0.09–0.22)	0.097(0.02–0.20)	0.189(0.12–0.30)
PTR	0.203(0.11–0.33)		0.126(0.09–0.17)	0.186(0.14–0.24)	0.115(0.08–0.15))	0.183(0.13–0.23)
MTR	0.402(0.32–0.50)	0.297(0.24–0.35)		0.190(0.14–0.23)	0.170(0.13–0.20)	0.207(0.16–0.25)
NNTR	0.351(0.28–0.44)	0.337(0.29–0.40)	0.354(0.29–0.41)		0.139(0.11–0.16)	0.187(0.15–0.22)
TATR	0.227(0.12–0.38)	0.258(0.21–0.30)	0.333(0.28–0.39)	0.295(0.26–0.34)		0.219(0.18–0.26)
STR	0.394(0.31–0.51)	0.332(0.28–0.39)	0.404(0.35–0.45)	0.340(0.29–0.39)	0.370(0.32–0.42)	

The summary statistics for the amplified markers (n = 12 loci) among the five clusters showed a higher mean number of alleles in NNTR and TATR (NNTRNa = 5.5 (SD 2.7) & TATRNa = 5.4 (SD 1.3), respectively) when compared with other three sub-populations (PTRNa = 4.3 (SD 1.5), STRNa = 4.9 (SD 1.1) and MTRNa = 3.4 (SD 1.1)). The highest observed heterozygosity was found in STR (Ho = 0.55 (SD 0.16)) followed by MTR (Ho = 0.50 (SD 0.26)), TATR (Ho = 0.49 (SD 0.20)), PTR (Ho = 0.45 (SD 0.22)) and NNTR (Ho = 0.39 (SD 0.17)), respectively. The mean allelic richness estimated from rarefaction method range from 3.35 (SD = 1.2) in MTR to 4.76 (SD = 2.2) in NNTR, with higher private alleles in STR and PTR (Supplementary Table 2).

### Gene flow and effective population size

The BAYESASS results showed very low and non-significant gene flow among the genetic subpopulations corroborating the distinct population structure patterns for dholes. The highest value for gene flow was from PTR to UKWLS and the lowest between UKWLS and NNTR (see Table 2 for details). Two independent, effective population size estimation approaches showed low values, ranging from 6–16.3 across the dhole subpopulations, suggesting potential inbreeding (See Table 3). Based on our data on unique individuals from each subpopulation and calculated effective population sizes, we found a very skewed ratio of Ne/N in TATR (0.16), NNTR (0.18) and MTR (0.17) but a balanced value in STR (0.29) and PTR (0.35) (Supplementary Table 4). The estimates should be taken with caution due to the use of surrogate census population size. The inbreeding coefficient (Fis) value ranged between 0.005–0.296 (Table 3) among the subpopulations. Careful investigation revealed a pattern where populations with lower Fis showed higher effective population sizes.

**Table 2:** Results of gene flow analysis using BAYESASS.

A(horizontal row)/B(vertical row)	UKWLS	PTR	MTR	NNTR	TATR	STR
UKWLS	**0.6891(0.0207)**	0.1647(0.0462)	0.0223(0.0207)	0.0228(0.0215)	0.0788(0.0402)	0.0223(0.0205)
PTR	0.0085(0.0082)	**0.9382(0.0234)**	0.0212(0.0160)	0.0093(0.0092)	0.0117(0.0110)	0.0110(0.0106)
MTR	0.0080(0.0079)	0.0133(0.0128)	**0.9490(0.0215)**	0.0095(0.0092)	0.0121(0.0114)	0.0081(0.0080)
NNTR	0.0038(0.0038)	0.0121(0.0097)	0.0144(0.0095)	**0.9525(0.0133)**	0.0061(0.0056)	0.0111(0.0064)
TATR	0.0039(0.0038)	0.0236(0.0114)	0.0062(0.0060)	0.0046(0.0045)	**0.9578(0.0140)**	0.0039(0.0038)
STR	0.0058(0.0057)	0.0086(0.0077)	0.0058(0.0057)	0.0062(0.0060)	0.0070(0.0065)	**0.9666(0.0136)**

The posterior distribution values of migraion rates (m) with 95% CI is presented. Bold values represent the proportions of individuals derived from their source population. The direction of gene flow is from A to B in this table.

**Table 3:** Estimates of effective population sizes (Ne) (C.I.-95%) and inbreeding coefficient (Fis) values at five sampled areas from LD approach.

	PTR (n = 33)	MTR (n = 35)	NNTR (n = 90)	TATR (n = 84)	STR (n = 54)
Ne estimator	11.8 (7.8–18.6)	6.0 (2.9–10.4)	16.3 (12.7–21.0)	13.5 (8.9–20.0)	16.0 (10.6–25.1)
LDNE	12.2 (8.3–18.6)	6.0 (3.1–9.7)	16.6 (13.4–20.7)	11.8 (9.7–14.4)	18.4 (13.3–26.3)
Fis value	0.223	0.29	0.074	0.071	− 0.0005

### Detection of demographic changes

Both qualitative analyses revealed signatures of population decline in the dhole subpopulations. BOTTLENECK results showed significant heterozygosity excess for NNTR, TATR and STR populations under all the three mutation models (IAM, TPM, SMM), suggesting a loss of rare alleles during a possible population decline (Supplementary Table 5). Similarly, the Garza-Williamson index showed low values (compared to M_cric_ 0.68 for stable populations) in all populations (M-ratio_PTR_- 0.27611 (SD 0.12); M-ratio_MTR_- 0.35809 (SD 0.24); M-ratio_NNTR_- 0.33504 (SD 0.15); M-ratio_TATR_-0.28588 (SD 0.12) and M-ratio_STR_-0.34592 (SD 0.09), indicating signals of population decline.

**Figure 3:**
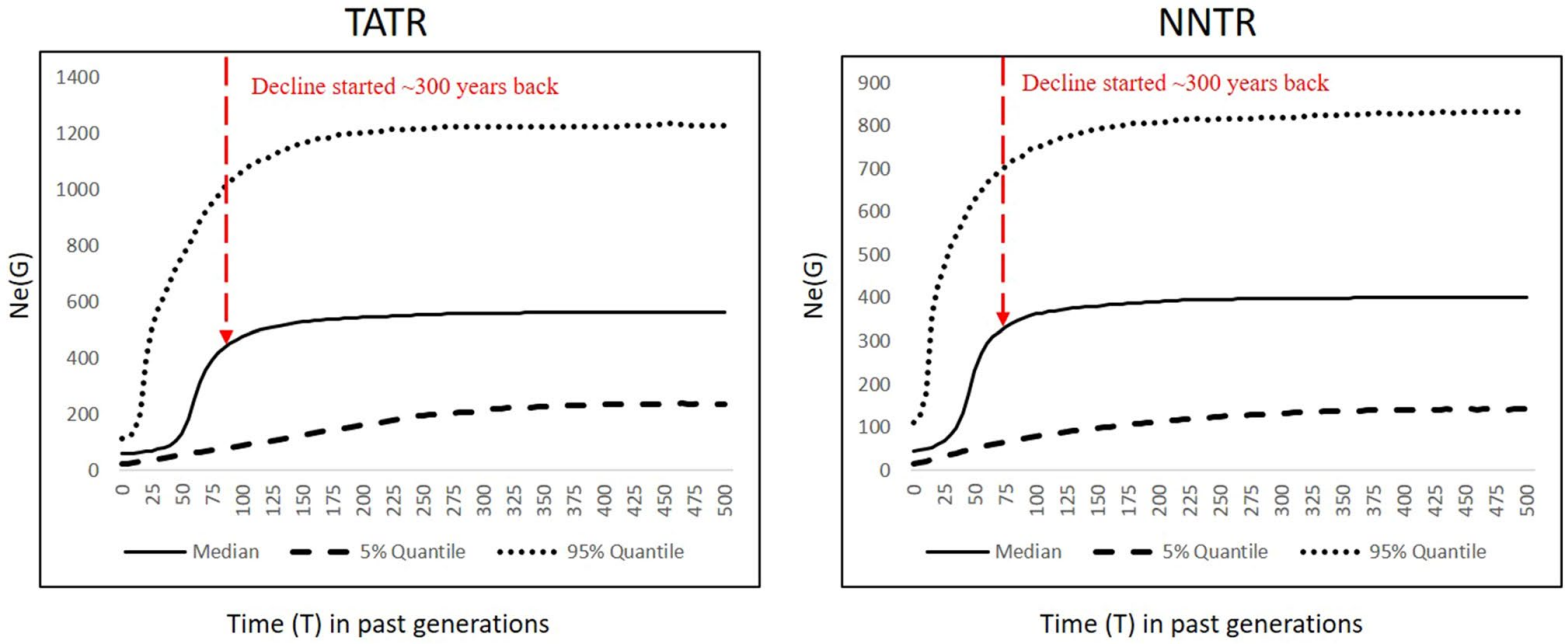
Demographic history of dholes in TATR and NNTR, Maharashtra through quantitative VarEff approach. Demographic changes have been presented as posterior distribution (median estimates) of the effective population sizes (0–500 generations ago) based on simulations with 12 microsatellite loci data from TATR (n = 84 individuals) and NNTR (n = 90 individuals). The decline timing has a median distribution value of ~ 300 years from present. The figure was generated using the package “VarEff” in R studio R Core Team (2019). R: A language and environment for statistical computing. R Foundation for Statistical Computing, Vienna, Austria.(https://www.R-project.org/).

The quantitative VarEff approach showed a steep decline in the effective population size in both NNTR and TATR subpopulations. Results indicate a decline of 77–85% in dhole effective population size for NNTR and TATR subpopulations. The timing of this decline was quantified at ~ 60–90 generations ago, making it about 300–450 years before present (with five years of generation time for dholes) (Fig. 3). The posterior distribution of estimates of Log(Ne) in the past 500 generations are provided in Supplementary Fig. 4. The current effective population size ranged between 21–114 (median 58) for TATR and 14–110 (median 45) for NNTR at a 95% confidence interval.

## Discussion

Generating detailed information on dhole population parameters at the landscape scale is highly challenging due to their obligate forest dependency, elusive nature and sensitivity towards anthropogenic activities. Therefore, dhole research has generally been focused on local habitat scales (for example, behavioural studies by^20,22,32^, population patterns by^28^) or at their distribution and occupancy using standard approaches^33^. The present study is perhaps the most exhaustive primary information on dhole genetics and population patterns across the species distribution to the best of our knowledge. We adopted a multidisciplinary approach involving field sampling, genetic information, and multivariate & Bayesian analytical frameworks to address spatial genetic patterns and demography of the largest dhole population in the central Indian landscape. This region is currently facing significant changes in land use pattern from rapid urbanisation, expanding agriculture, infrastructure development, acquisition of minerals and economic growth^34,35^. Our results from this study thus have important conservation/management implications for dholes and their habitat.

Firstly, multiple genetic analyses with landscape-scale microsatellite data reveal five distinct dhole genetic clusters. Out of six sampled areas, the five major tiger reserves (MTR, PTR, TATR, NNTR and STR) represents the five clusters. While the UKWLS a known connecting habitat between PTR and TATR^36^ showed a mixture of genetic signals from the four major clusters (TATR, NNTR, MTR and PTR). The genetic clusters were clearly separated with very few mixed genetic signals in UKWLS (Fig. 1a). Despite any assignment cluster for UKWLS, it was included further in connectivity analysis due to its strategic geographical location in the landscape acting as a connecting link^36^. However, such pattern is not surprising as dholes are highly forest-dependent and short-range dispersers^33^; this situation highlights their vulnerability in the current scenario of continuing land-use change and urbanization. Similar group-living species have been reported to show genetic differentiation due to their adaptations to specific habitat, group cohesion and local philopatry^37^. For example, social canids such as gray wolf, African wild dog have also shown higher genetic differentiation in fragmented landscapes^38^. Contrary to this, other co-occurring large carnivores (tiger^39^; leopard^40^) and omnivore (sloth bear^41^) showed much less genetic differentiation, possibly due to longer dispersal capabilities resulting in higher gene flow. Given the small population size of dholes across their range^14^ , and possible genetic differentiation (based on the results of this study), the species face potential demographic impact^19,42^ Kamler et al.^14^ suggested that dholes require an area five times larger than tiger for long-term viability, which seems to be improbable in the current scenario. Thus, it will be critical to maintain population connectivity through corridor restoration and facilitate gene flow at the landscape scale. The NNTR and TATR clusters show a comparatively higher number of first-generation migrants with respect to other sub-populations. One of the major reasons behind such a pattern can be the larger pack size owing to low tiger density in NNTR^43^ and higher turnover in TATR due to higher tiger density. In low tiger density areas, breeding opportunities reduce because of the larger pack size, required to suppress the recovery of top-predator, while in high tiger density areas, immigrants often fail to establish themselves hence, contribute less to the gene pool. These dynamics of pack size and top-predator density influence the genetic structure of a population.

Our demography analyses with two qualitative approaches indicate strong decline in dhole population size for all five genetic subpopulations, as expected from fragmented and small, isolated populations^44^. The quantitative approach with VarEff revealed a 77–85% decline in NNTR and TATR dhole populations, respectively. The magnitude of decline for dholes corroborates with other co-occurring large carnivores in this landscape. For example, earlier studies have shown a 90% and 98% decline in leopard and tiger population in the central Indian region, respectively^45,46^. The relatively less decline for dholes compared to other larger carnivores is possibly due to fewer demands of dholes as trophies/illegal wildlife trade. Tiger and leopard body parts (pelt, bones, claw, meat, fat, whisker ) are highly sought products in trans-national illegal wildlife trade^47^, whereas dhole populations have faced historical pressures from bounty hunting and human persecutions as vermins during British colonial period^14^. However, it is difficult to validate these decline patterns with other information as no robust quantitative data on actual population size (both historical and current) is available^48^. Another important finding is the relatively old timing of decline for dholes. Our results suggest a median dhole decline timing of ~ 300 years in both NNTR and TATR, much older than tiger/leopard decline timing in central India (tiger- decline ~ 200 years ago^46^, leopard- decline ~ 125 years ago^45^). This could be explained by a combination of habitat loss driven population decline in historical times followed by hunting during the British era^29^. The study done on global land-use change over the last 300 years have also estimated a forest loss of 40% for the Indian sub-continent during the last century^49,50^. Sharma et al.^51^ showed that this landscape had experienced a major change in land-use patterns during the last 300 years, leading to ~ 77% loss of forested habitats to agricultural area and urbanization. Rangarajan^52^ also reported severe fragmentation of historically contiguous habitats of the Central Indian Highland during the last few centuries. Subsequently, over-exploitation of teak started during the early British period (1750–1990) for navy and railway lines which further resulted in the conversion of forests into commercial plantation by large scale clearing^53,54^, thereby further reducing the available habitats for dholes. We feel that such drastic changes possibly had severe impacts on the population size of obligate forest-dwelling dholes. Although comprehensive data on the exact effects of such large-scale habitat loss on dhole population size is lacking, this available information suggests that continuing habitat fragmentation starting since last 300 years coupled with massive hunting pressure during colonial bounty-hunting rules resulted in dhole population decline over a longer time..

One of the most important aspect of this study is the assessment of inbreeding status (Fis value) and effective population size (Ne) of dholes which are critical population parameters, and summarise the history of any population^55^. For both NNTR and TATR populations (relatively higher population size compared to the other areas), the Ne is approximately 20% of the total population (Ne/N ratio of 0.16 and 0.18 for TATR and NNTR, respectively) which is similar to 0.11 across different taxa^56^. Such low values for Ne are not unusual and have been earlier described in social animals with dominance hierarchy (for example, lions^57^, African wild dog^58^, dwarf mongooses^58^) as well as in endangered species with small population sizes^59^. However, the Ne values obtained from VarEff were comparatively higher and probably realistic than the linkage disequilibrium based approach as this approach can substantially underestimate the Ne in inbred populations^43,57,58,60^. The number of individuals observed in each population could be related to the sampling size which is a limitation in our study due to unavailability of population estimates. We have only used the population size from this study as a surrogate to calculate the Ne/N ratio which is independent of Ne calculation. In addition, we also found out that inbreeding coefficient value (Fis) were different for each population and indicated an inverse relationship with Ne. For example, dhole data from MTR showed the highest Fis value and lowest Ne, whereas STR showed the lowest Fis and high Ne value. This pattern makes sense for a species with social dominance hierarchy where only the dominant member of the pack has highest mating opportnities (thus low Ne) and will have more inbred individuals in a small group or population (high Fis).

Finally, our results from this study also showed that relatively large dhole populations such as NNTR and TATR still retain reasonable high genetic variation despite the severe decline and strong population structure. The genetic variations of NNTR and TATR are comparable with other social canid species such as African wild dog from Kruger National Park^61^. However rest of the populations (need urgent management interventions (possibly in the form of translocations as well as better habitat connectivity) to increase the genetic variation and ensure the future survival of the populations in this landscape as a whole. We acknowledge the chances of bias in our result of individuals identified with low dhole recaptures, which can be correlated with complex interactions between our specific sampling strategy and relatively low amplification success rates from field-collected faeces. Since our field sampling strategy focused on maximum coverage within six protected areas of central Indian dhole distribution and was conducted only once, during which we surveyed all possible latrine sites and collected only fresh samples for DNA analyses. Because of this, we might have missed recapturing the same individuals, thus giving us low recapture rates.

This can be dealt with a genetic capture recapture method in future with more intensive sampling strategy. The high difference in the expected and observed heterozygotes could be a complex combination of species-population marker scenario as also found in other canid (gray wolf, coyote, golden jackal) studies where cross-species dog primers were used^62,63^.

## Conclusion

Despite sharing most of their current range within India with tiger and leopard, ecological information on dholes are still inadequate for appropriate management planning. Historical information and our quantitative data indicate that the last 300 years has brought drastic reductions in dhole distribution and population size^64^ and some cases local extinction^14^. Initiation of relentless tiger conservation efforts since the 1980s has helped the species to survive in most of its existing range^65^, but unlike tigers, the dhole population trend continues to decrease globally. Currently, the major strongholds of the species are the Western Ghats (Karnataka) and central India (Maharashtra)^48^, where focused conservation efforts are urgently required. With the ongoing habitat fragmentation scenario, dhole-specific threats (habitat loss, prey depletion, disease transmission, human persecution ) must be addressed to ensure the long-term persistence of the species. We hope that the results and suggestions from this study will lead to generation of critical information on dhole genetics from Central Indian Landscape which will aid in understanding the effects in the similar landscape across dhole distribution range. We hope the information will help in developing informed strategies for conservation.

## Methods

### Research permissions and ethical considerations

Permissions for fieldwork and sampling were granted by the Maharashtra Forest Department (Permit No. 09/2016). This work did not require any approval from the ethical committee due to its non-invasive nature.

### Study area and sampling

We conducted this study in Maharashtra, which retains one of the major dhole populations in central India. We sampled major known dhole habitats across the state, covering five tiger reserves: Pench Tiger Reserve (PTR), Melghat Tiger Reserve (MTR), Sahyadri Tiger Reserve (STR), Tadoba-Andhari Tiger Reserve (TATR) and Navegaon-Nagzira Tiger Reserve (NNTR) (Fig. 1a). Apart from these areas, we also sampled surrounding regions of Umred-Karandhla Wildlife Sanctuary (UKWLS). This mosaic of the tiger reserves and surrounding regions is already established as tiger corridors^36^, making it important to see if obligate forest-dwelling dholes are also using the same corridors. STR is completely disconnected from all other sites (Fig. 1a). The remaining areas form a complex network of habitat patches, where the remaining sites are known to have varying degrees of habitat connectivity^36^. MTR is an exception to this complex as it does not share direct connectivity with the tiger reserves in this complex. All of these areas are characterized by dry deciduous to moist deciduous forests^66^.

We sampled the entire region between January 2016 to April 2019, covering PTR (257.3 km^2^), MTR (1500.49 km^2^), NNTR (152.8 km^2^), TATR (627.5 km^2^), STR (1166 km^2^) and UKWLS (189 km^2^), Maharashtra. Each site was sampled intensively once for dhole scats. Through foot and vehicle surveys, extensive scat sampling resulted in 623 scats from 82 latrine sites across all seven study areas. We only collected fresh samples during field surveys, where one bolus/ scat was stored in butter paper following approaches described in Biswas et al.^67^. We also recorded the GPS coordinates and other associated field information (substrate, track marks) for each sample. In the field, the samples were temporarily stored in a large box containing silica gel. In some cases, we sprayed a small amount of absolute ethanol to minimize fungal growth^68^. The samples were kept in the field for a maximum period of 10 days. Once transferred to the laboratory, all the scat samples were stored in a − 20 °C freezer till further processing.

### DNA extraction and species identification

We performed DNA extraction from all field-collected scats using already established approaches described in Modi et al.^27^. In brief, we either swabbed twice (samples with no dust) or scraped (samples covered with dust) the top layer of the samples with sterile swabs or blade, respectively. They were lysed overnight in a lysis buffer at 56 °C, and extraction was performed following QIAamp DNA Tissue Kit (Qiagen Inc, Hilden, Germany) protocol. Final elution was performed twice in 100 μl of 1X TE buffer, and the DNA was stored at − 20 °C for long-term use.

We conducted molecular species identification using dhole-specific mitochondrial DholespID-F/R primers described in Modi et al.^27^. PCR reactions were performed in 10 µL volumes with 4 µL of hot-start taq mix (Qiagen Inc, Hilden, Germany), 4 µM BSA, 0.5 µM of primer mix and 3 µL of DNA extract. PCR conditions included an initial denaturation (95 °C for 15 min); 50 cycles of denaturation (94 °C for 30 s), annealing (50 °C for 30 s) and extension (72 °C for 35 s); followed by a final extension (72 °C for 10 min). Negative and extraction controls were included to monitor contaminations. Species ascertainment was done through visualization of dhole-specific bands (236 bp) in 2% agarose gel. All the experiments were conducted in Conservation Genetics Lab in Wildlife Institute of India, Dehradun.

### Individual identification

For individual identification from the confirmed dhole scats, we used the earlier validated 12 microsatellite loci panel described in Modi et al.^26^ (Supplementary Table 1). We performed PCR reactions in 10 μl reaction volumes containing 4 μl of Multiplex master mix (QIAGEN Inc., Hilden, Germany), 4 μM (2.5 μl) BSA, 0.5 μM of primer mix and 3 μl of DNA extract with PCR conditions including initial denaturation (95 °C for 15 min); 50 cycles of denaturation (94 °C for 30 s), annealing (50 °C for 30 s) and extension (72 °C for 35 s); followed by a final extension (72 °C for 10 min)^26^. Negative and extraction controls were included to monitor contaminations. Amplified products were mixed with HiDi formamide and LIZ 500 size standard (Applied Biosystems, California, United States) and genotyped in an ABI genetic analyzer (Applied Biosystems, California, United States). We scored the fragment lengths manually using the same reference sample and following stringent criteria described in Modi et al.^26^. All samples were genotyped three independent times to ensure good data quality for subsequent analyses. We have also included 101 individual genotypes from our previous study^26^ collected from five protected areas (MTR, TATR, PTR, NNTR, UKWLS) along with the newly generated data for further analysis.

### Data analyses

To generate the best quality data for analyses, we prepared consensus genotypes of each locus following the multiple tube approach combined with quality index protocol described in Modi et al.^26^. We only considered the genotypes, which produced data for atleast seven out of 12 loci in the consensus^26^ The quality index threshold of 0.66 per loci, while the mean quality index of 0.75 across loci was set for the samples to be considered for downstream analyses. We used MICROCHECKER v 2.2.3^69^ to determine large allele dropouts as well as genotyping error estimation module of GIMLET^26,70^ to calculate overall genotyping error rates (allelic dropout and false alleles). We used FreeNa (Chapuis & Estoup, 2007) to determine the frequency of null alleles (NAs), which estimates the NA frequency using EM algorithm (Dempster, Laird & Rubin, 1977). We removed all genetic recaptures using the identity analyses module of CERVUS^71^, allowing up to two mismatches and calculated the cumulative *P*_ID(unbiased)_ (probability of identity) and *P*_ID(sibs)_ value^72^ using GIMLET^73^. We estimated the allelic richness using the rarefaction approach in HP-RARE considering the uneven sample size of populations. We used GENPOP and ARLEQUIN^74^ to check deviations from Hardy–Weinberg equilibrium (HWE) and linkage disequilibrium (LD).We also conducted relatedness test using the pairwise relatedness estimators TrioML, QGM, LRM and DyadML incorporated in COANCESTRY v1.0.1.8 to avoid any bias due to related individuals. Both TrioML and Dyad ML considered genotyping errors and had the smallest variance.

### Inferring population structure

To infer any possible genetic structure of dholes across the sampled areas, we used a combination of Bayesian clustering and multivariate analyses. These analyses were conducted for only those populations with data from at least ten different individuals.

We implemented the Bayesian clustering approach through program STRUCTURE v.2.3.4^75^, where 10 independent runs were performed for a range of population values (K = 1 to 10) with 100,000 burnin and 500,000 iterations. The models were run with admixture models considering correlated allele frequency. The optimal number of clusters was determined by the deltaK approach^76^ implemented in STRUCTURE HARVESTER^77^. The admixture proportion of individuals over ten replicates were averaged using CLUMPAK^78^. The ancestry coefficient of the individuals produced by STRUCTURE was interpolated on a map using the R package tess3r^79^.

Further, we used the program Discriminant Analysis of Principal Component (DAPC)^80^ to identify genetic clusters in our data. This is a multivariate analytical approach where no spatial information is required, and the population does not require to be under Hardy–Weinberg Equilibrium^80,81^. The genetic data is transformed into principal components, followed by clustering using the discriminant function to define a group of individuals with minimum within-group variation and maximum between-group variations. We conducted the analyses using adegenet package 2.1.1 in R studio 1.1.453 (R Development Core Team 2018), where an optimal number of clusters was determined through the Bayesian Information Criterion^80^, and number of clusters was assessed using find. clusters *dapc function in R.*

Finally, we used another multivariate method implemented in program spatial Principal Component Analysis (sPCA) that investigates cryptic spatial patterns of genetic variability using georeferenced multilocus genotypes^82^. sPCA incorporates the spatial information along with the genotype data to ascertain local and global patterns of variations^83^. The global pattern (positive autocorrelation) would differentiate between two spatial groups, whereas the local pattern (negative autocorrelation) would determine the genetic differences among neighbours. The analysis was carried out using the nearest neighbour as the connection network. The variance was plotted against spatial autocorrelation (Moran’s I)^84^ to estimate any spatial structure in the genetic data visually. We used the Monte Carlo test with 10,000 iterations to statistically test global and local spatial structure.

### Genetic differentiation among dhole populations

We estimated genetic differentiation through different indices (G’st and Jost D)^85,86^ using the R package DiveRsity 1.9^87^ in R studio 3.1. We used both the differentiation indices to elucidate the asymmetric migration^88^ and differentiation among the sub-populations^89,90^.

### Assessment of gene flow among different subpopulations

We used a Bayesian approach implemented in BAYESASS ver. 3.0.3^91^ to infer the contemporary migration rate (m) among the detected subpopulations. This approach detects recent, low immigration rates in a population based on the genotype disequilibrium relative to the sampled populations without assuming HW equilibrium within the populations. The run parameters included 3 × 10^6^ iterations and 10^6^ burn-in with sampling at every 2000 iterations. Delta values were adjusted to maintain an MCMC state change acceptance ratio of 20–40%. We averaged the results of multiple runs for best model fit, as indicated by the Bayesian deviance measure^92^.

### Effective population size (Ne)

We used the program Ne estimator v.2.01^93^ to estimate the *N*_*e*_ from genotype data. We used the random mating model and the following critical values (*P*_*crit*_): 0.05, 0.02 and 0.01 and jackknife 95% confidence interval for our analyses. We calculated the Ne for each subpopulation separately based on the number of putative clusters determined with a critical value of 0.02. We further used LDNe^94^, which also estimates the effective population size using the linkage disequilibrium approach with bias correction.

### Demography analyses

We used qualitative and quantitative approaches to determine past demographic patterns of dhole subpopulations based on population substructure analysis results. For qualitative analysis, we used two different summary statistics-based approaches to detect any signal of population decline in dholes. These approaches are the Ewens, Watterson, Cournet, and Luikart method implemented in program BOTTLENECK ver 1.2.02^95^ and the Garza-Williamson index/ M-ratio approach implemented in program ARLEQUIN^74^. For BOTTLENECK, simulations were performed under three mutation models: infinite allele model (IAM), single stepwise model (SMM), and two-phase model (TPM). For the TPM model, 30% of multi-step mutation events were allowed during the simulations. This method detects departures from mutation-drift equilibrium and neutrality, which can be explained by any departure from the null model, including selection, population growth, or decline. The Garza-Williamson index uses data on the frequency and the total number of alleles, and the allele size difference to investigate population decline.

Further, we used R package VarEff 1.2^96^ in the R software version 3.1 to quantify dhole demographic patterns. This approach uses a coalescent framework to estimate the variation in effective population size (Ne) from present to ancestral time and determines the time of population decline from genetic data. We performed the analysis assuming the stepwise mutation model (SMM)^97^ with a generation time of 5 years for Asiatic wild dogs^14^. We used the SMM model to describe the mutation process for microsatellites in a more wholesome way^98^. We considered a constant mutation rate of 3.5 × 10^−3^ per generation as described for canid microsatellites^99^ over the past 1000 generations. The models were set with parameter DMAXPLUS value of 4 and 6 from the allele frequency histograms (maximum distance observed with a frequency ≥ 0.005 at 4 and 6)^96^, along with prior values for Ne (parameter NBAR, range provided by theta), and the variances of the prior log-distributions for Ne (parameter VARP1, value of 3) and time intervals with constant population size (parameter VARP2, value of 3). The prior correlation coefficient between successive population sizes (parameter RHOCORN) was set to zero and Jmax value set at 2. The run parameters included the number batch to 10,000 length and space batch to 10, acceptance rate of 0.25 with a diagonal of 0.5. The demographic analyses were performed for only NNTR and TATR populations as they had adequate sample sizes.

## Supplementary Information

Supplementary Information.

## Data Availability

The microsatellite dataset used for different analyses in this study is available from the corresponding author on reasonable request.
