# Supplementary material for: Genetic analyses reveal demographic decline and population differentiation in an endangered social carnivore, Asiatic wild dog
Source: Sci Rep. 2021 Aug 12;11:16371. doi: 10.1038/s41598-021-95918-3 (PMC8361113; doi:10.1038/s41598-021-95918-3)
Supplement: Supplementary file 1 — Supplementary Information. [file 41598_2021_95918_MOESM1_ESM.docx]

**Genetic analyses reveal demographic decline and population differentiation in an endangered social carnivore, Asiatic wild dog**

Shrushti Modi^1^, Samrat Mondol^1^, Parag Nigam^1^, Bilal Habib^1*^

^1^ Wildlife Institute of India, Chandrabani, Dehradun, India 248001

^*^ Corresponding author: Bilal Habib, Ph.D. Wildlife Institute of India, Chandrabani, Dehradun, India 248001. Email: [bh@wii.gov.in](mailto:bh@wii.gov.in)

**Supplementary Table 1:** Summary statistics of the 12 microsatellite loci used for population genetic analyses of Asiatic wild dog in this study.

| **Locus** | **Motif** | **Na** | **Allele size difference** | **Genotyping error** | | | **Reference** |
| --- | --- | --- | --- | --- | --- | --- | --- |
|  |  |  |  | **ADO (per allele per locus)** | **FA (per allele per locus)** | **NA (%)** |  |
| **WD2201** | Tetra | 12 | 170-202 | 0.00 | 0.00 | 3.1 | Francisco et al (1996) |
| **PEZ6** | Tetra | 8 | 206-230 | 0.00 | 0.00 | 17.5 | Neff et al (1998) |
| **WD2140** | Tetra | 10 | 122-178 | 0.00 | 0.00 | 4.2 | Francisco et al (1996) |
| **AHT130** | Di | 8 | 98-112 | 0.30 | 0.00 | 1.2 | Holmes et al (1994) |
| **PEZ3** | Tetra | 9 | 110-146 | 0.00 | 0.24 | 16.4 | Neff et al (1998) |
| **WD2137** | Tetra | 12 | 156-180 | 0.00 | 0.14 | 21.3 | Francisco et al (1996) |
| **PEZ5** | Tetra | 10 | 150-254 | 0.00 | 0.13 | 15.2 | Neff et al (1998) |
| **CXX251** | Di | 6 | 128-136 | 0.00 | 0.00 | 10.6 | Ostrander (1995) |
| **WD2096** | Tetra | 7 | 93-115 | 0.00 | 0.10 | 19.8 | Francisco et al (1996) |
| **CXX279** | Di | 7 | 123-135 | 0.00 | 0.23 | 13 | Ostrander (1995) |
| **WD2001** | Tetra | 6 | 134-142 | 0.173 | 0.00 | 5.8 | Francisco et al (1996) |
| **CXX30** | Di | 10 | 122-142 | 0.00 | 0.00 | 3 | Ostrander (1995) |
| **Mean** |  | 8.75 |  | 0.040 | 0.071 | 10.9 |  |

Na=number of alleles, ADO= Allele Dropout, FA= False alleles, NA= Null alleles

**Supplementary Table 2:** Subpopulation-wise summary statistics of 305 dholes at twelve microsatellites across Maharashtra, India.

| Protected areas | PTR (n=33) | | | | MTR (n=35) | | | | NNTR (n=90) | | | | TATR (n=84) | | | | STR (n=54) | | | | |
| --- | --- | --- | --- | --- | --- | --- | --- | --- | --- | --- | --- | --- | --- | --- | --- | --- | --- | --- | --- | --- | --- |
| Locus | Na | Ar | Ho | He | Na | Ar | Ho | He | Na | Ar | Ho | He | Na | Ar | Ho | He | Na | Ar | Ho | He |  |
| CXX30 | 3 | 3.0 | 0.76 | 0.61 | 1 | 1.0 | 0.0 | 0.0 | 3 | 2.2 | 0.0 | 0.06 | 6 | 5.2 | 0.12* | 0.35 | 5 | 4.0 | 0.31 | 0.40 |  |
| CXX279 | 3 | 2.9 | 0.21 | 0.35 | 3 | 2.9 | 0.17 | 0.22 | 4 | 3.4 | 0.32* | 0.67 | 4 | 3.7 | 0.18 | 0.29 | 5 | 4.8 | 0.65 | 0.61 |  |
| WD2001 | 2 | 2.0 | 0.21 | 0.50 | 3 | 2.7 | 0.63 | 0.51 | 3 | 2.3 | 0.16 | 0.23 | 6 | 4.4 | 0.38* | 0.60 | 4 | 3.8 | 0.48 | 0.46 |  |
| WD2096 | 3 | 3.0 | 0.76 | 0.61 | 2 | 2.0 | 0.57 | 0.41 | 3 | 3.0 | 0.43 | 0.45 | 4 | 3.1 | 0.76 | 0.50 | 6 | 4.6 | 0.61 | 0.53 |  |
| AHT130 | 6 | 5.9 | 0.27* | 0.71 | 4 | 3.7 | 0.60 | 0.62 | 6 | 5.4 | 0.40 | 0.57 | 5 | 2.8 | 0.46 | 0.50 | 6 | 5.9 | 0.31* | 0.67 |  |
| CXX251 | 3 | 2.9 | 0.27* | 0.54 | 3 | 3.0 | 0.14* | 0.57 | 5 | 4.5 | 0.39* | 0.62 | 4 | 3.3 | 0.39* | 0.59 | 4 | 3.7 | 0.69 | 0.56 |  |
| WD2140 | 5 | 4.8 | 0.33 | 0.56 | 4 | 3.9 | 0.51 | 0.58 | 4 | 3.7 | 0.39 | 0.46 | 6 | 5.2 | 0.46 | 0.64 | 3 | 3.0 | 0.33* | 0.66 |  |
| PEZ3 | 7 | 6.1 | 0.58 | 0.67 | 4 | 3.9 | 0.66 | 0.67 | 6 | 5.9 | 0.59 | 0.79 | 7 | 6.3 | 0.65 | 0.78 | 5 | 4.5 | 0.65 | 0.63 |  |
| PEZ5 | 4 | 3.9 | 0.15* | 0.47 | 3 | 3.0 | 0.17* | 0.47 | 4 | 3.5 | 0.12 | 0.27 | 3 | 2.4 | 0.17 | 0.21 | 3 | 2.8 | 0.24* | 0.52 |  |
| PEZ6 | 5 | 5.0 | 0.27* | 0.77 | 4 | 4.0 | 0.54 | 0.70 | 6 | 5.2 | 0.36* | 0.66 | 6 | 4.7 | 0.42 | 0.60 | 5 | 4.9 | 0.26* | 0.65 |  |
| WD2201 | 7 | 6.9 | 0.64 | 0.73 | 6 | 5.9 | 0.66 | 0.69 | 10 | 8.2 | 0.23* | 0.65 | 8 | 7.5 | 0.52 | 0.75 | 7 | 6.5 | 0.41 | 0.74 |  |
| WD2137 | 4 | 4.0 | 0.42 | 0.64 | 4 | 3.8 | 0.63 | 0.55 | 12 | 9.4 | 0.42 | 0.68 | 6 | 5.8 | 0.55 | 0.66 | 6 | 4.9 | 0.50 | 0.65 |  |
| Mean (SD) | 4.3  (1.5) | 4.23(1.5) | 0.45  (0.22) | 0.60  (0.11) | 3.4  (1.1) | 3.35(1.2)) | 0.50  (0.26) | 0.50  (0.20) | 5.5  (2.7) | 4.76(2.2) | 0.39  (0.17) | 0.51  (0.17) | 5.4  (1.3) | 4.57(1.5) | 0.49  (0.20) | 0.54  (0.17) | 4.9  (1.1) | 4.49(1.0_ | 0.55  (0.16) | 0.59  (0.09) |  |

Na=number of alleles per locus., Ar= standardized number of alleles, H_o_= Observed heterozygosity, H_e_= Expected heterozygosity.
*Loci not in Hardy-Weinberg Equilibrium in the respective populations.

Supplementary Table 3: Mean and variance (in bracket) of four relatedness estimators available in Coancestry. TrioML has the lowest variance for each sub-population. Both TrioML and DyadML calculates positive related estimates between zero and one..

| Protected area | **TrioML** | QGM | LRM | **DyadML** |
| --- | --- | --- | --- | --- |
| PTR | **0.09 (0.02)** | -0.028(0.09) | -0.037(0.06) | **0.11 (0.03)** |
| MTR | **0.12 (0.04)** | -0.033(0.13) | -0.037(0.09) | **0.14 (0.05)** |
| NNTR | **0.10 (0.03)** | -0.003(0.10) | -0.011(0.06) | **0.12 (0.04)** |
| TATR | **0.10 (0.02)** | -0.002(0.09) | -0.015(0.05) | **0.12 (0.03)** |
| STR | **0.10 (0.02)** | -0.016(0.10) | -0.025(0.06) | **0.13 (0.03)** |

Supplementary Table 4: Samples collected and individual identified from each tiger reserve

| Protected area | Samples collected | Individual identified (N) |
| --- | --- | --- |
| UKWLS | 23 | 9 |
| PTR | 56 | 33 |
| MTR | 58 | 35 |
| NNTR | 194 | 90 |
| TATR | 180 | 84 |
| STR | 112 | 54 |

**Supplementary Table 5: Sign test results from BOTTLENECK analysis under IAM, TPM and SMM mutation model. P-values are in bracket and significant results are in bold.**

| Population | Sign Test: Number of loci with heterozygosity excess (probability) |  |  |
| --- | --- | --- | --- |
|  | IAM | TPM | SMM |
| PTR | **6.59(0.007)** | 6.96(0.383) | 7.02(0.374) |
| MTR | **5.89(0.010)** | 6.43(0.260) | 6.44(0.506) |
| NNTR | 6.68(0.546) | **7.05(0.019)** | **7.14(0.016)** |
| TATR | 6.81(0.162) | **7.12(0.003)** | **7.09(0.003)** |
| STR | 6.85(0.056) | **7.07(0.003)** | **7.18(0.002)** |

**Supplementary Figure 1. (a)** Ln(K) plot with variance of the 10 runs for each K **(b)** delta K graph using Evanno’s coefficient to determine the value of K **(c)** BIC (Bayesian Inference Criterion) to determine the number of cluster for DAPC(d) Bar plot represents the cluster from K=2 to K=4 obtained from STRUCTURE where each color corresponds to a cluster and the vertical bar corresponds to an individual.


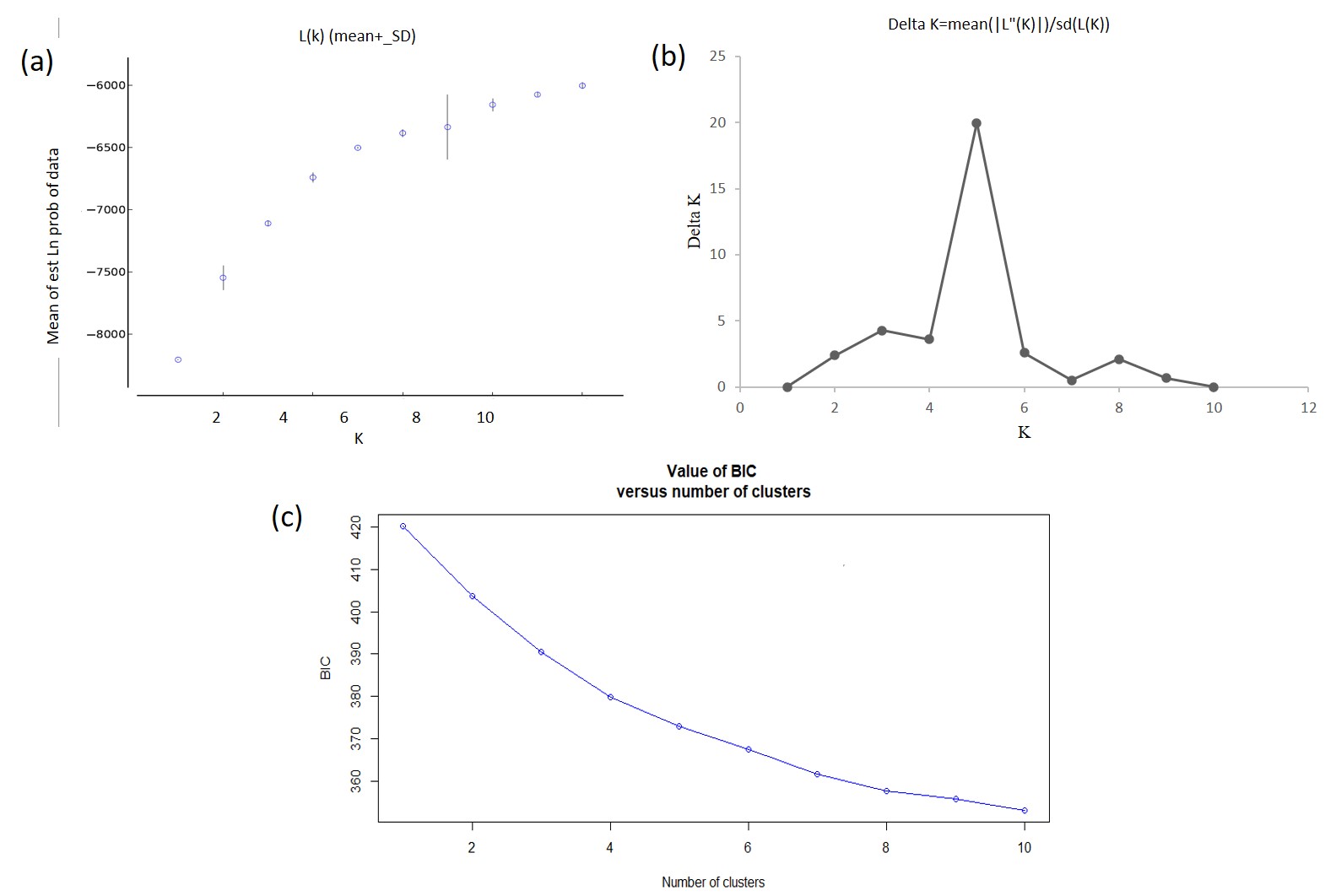


(d)


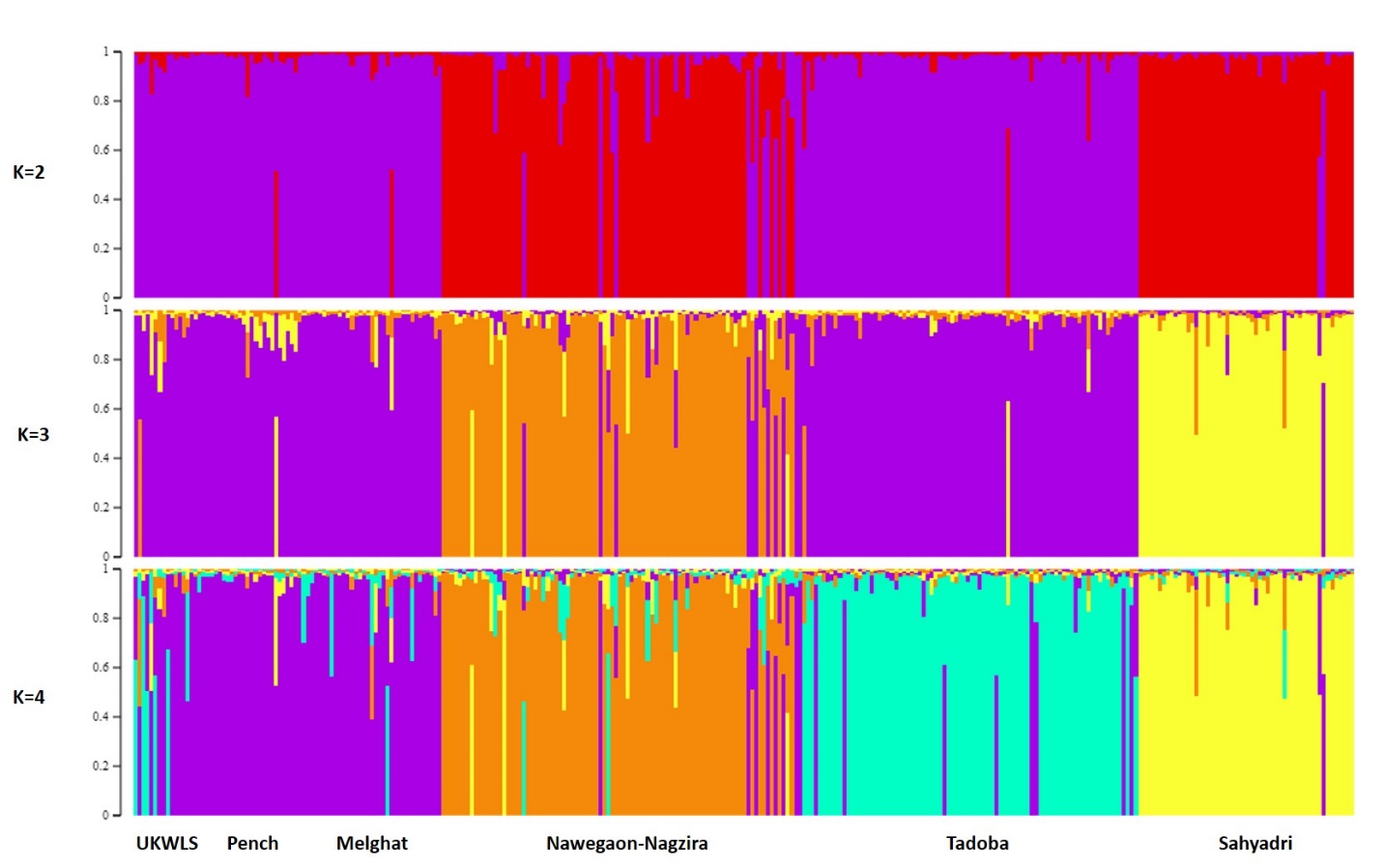


**Supplementary Figure 2:**  Results of Eigen value test for sPCA analysis. The global differentiation is significant for dholes when compared to the local differentiation. The figure was generated using the package “adegenet” in R studio R Core Team (2019). R: A language and environment for statistical computing. R Foundation for Statistical Computing, Vienna, Austria. URL https://www.R-project.org/.

**
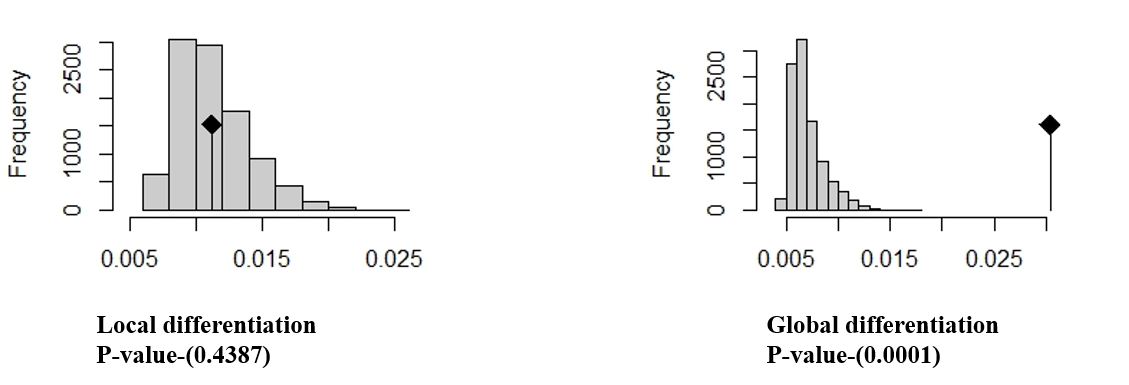
**

**Supplementary Figure 3:** Figures showing the Eigenvalues and spatial weightings to represent the connection network and differentiation scores for the sampled dhole individuals (n=305) in this study. The first plot shows the connection network that was used to define spatial weightings (CN=6). The second plot is a local interpolation of scores using grey levels, with contour lines; more closer contour lines represent steepest differentiation. The third plot uses different sizes of squares to represent different absolute values: white for stronger negative values and black for positive values. The fourth plot is a variant using grey levels where smaller, grey (light grey = positive, dark grey = negative) squares are less strongly differentiated. Lower two eigenvalue plots and the screeplot indicate that the first three axes are most important in explaining genetic variation. The screeplot plots the relationship between variance explained by each axis and spatial autocorrelation (Moran’s I) within each axis). The figure was generated using the package “adegenet” in R studio R Core Team (2019). R: A language and environment for statistical computing. R Foundation for Statistical Computing, Vienna, Austria. URL https://www.R-project.org/.


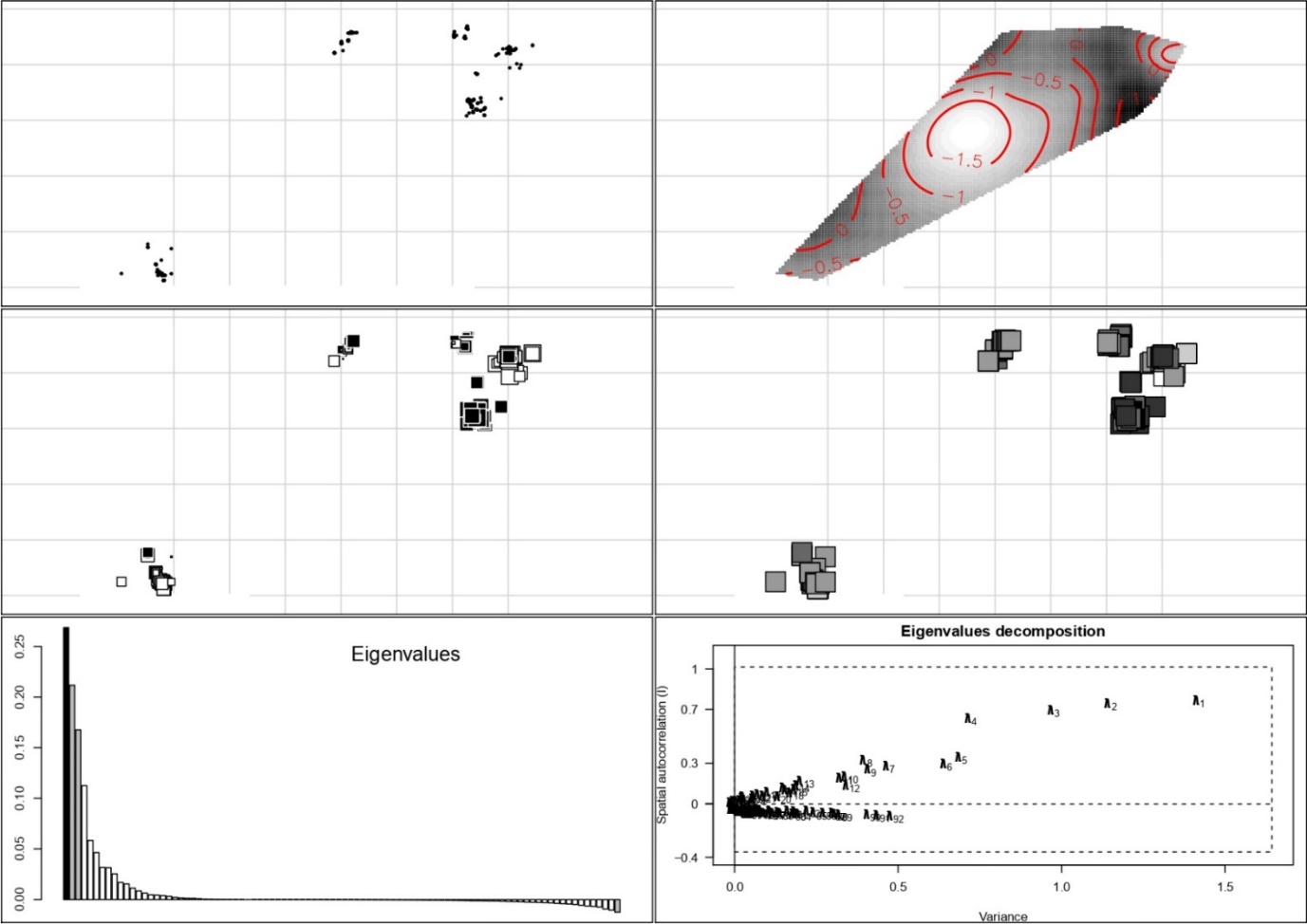


**Supplementary Figure 4:** The posterior distribution of estimates of Log(Ne) in the past 500 generations analyzed by VarEff TATR and NNTR populations respectively. The figure was generated using the package “VarEff” in R studio R Core Team (2019). R: A language and environment for statistical computing. R Foundation for Statistical Computing, Vienna, Austria. URL https://www.R-project.org/.


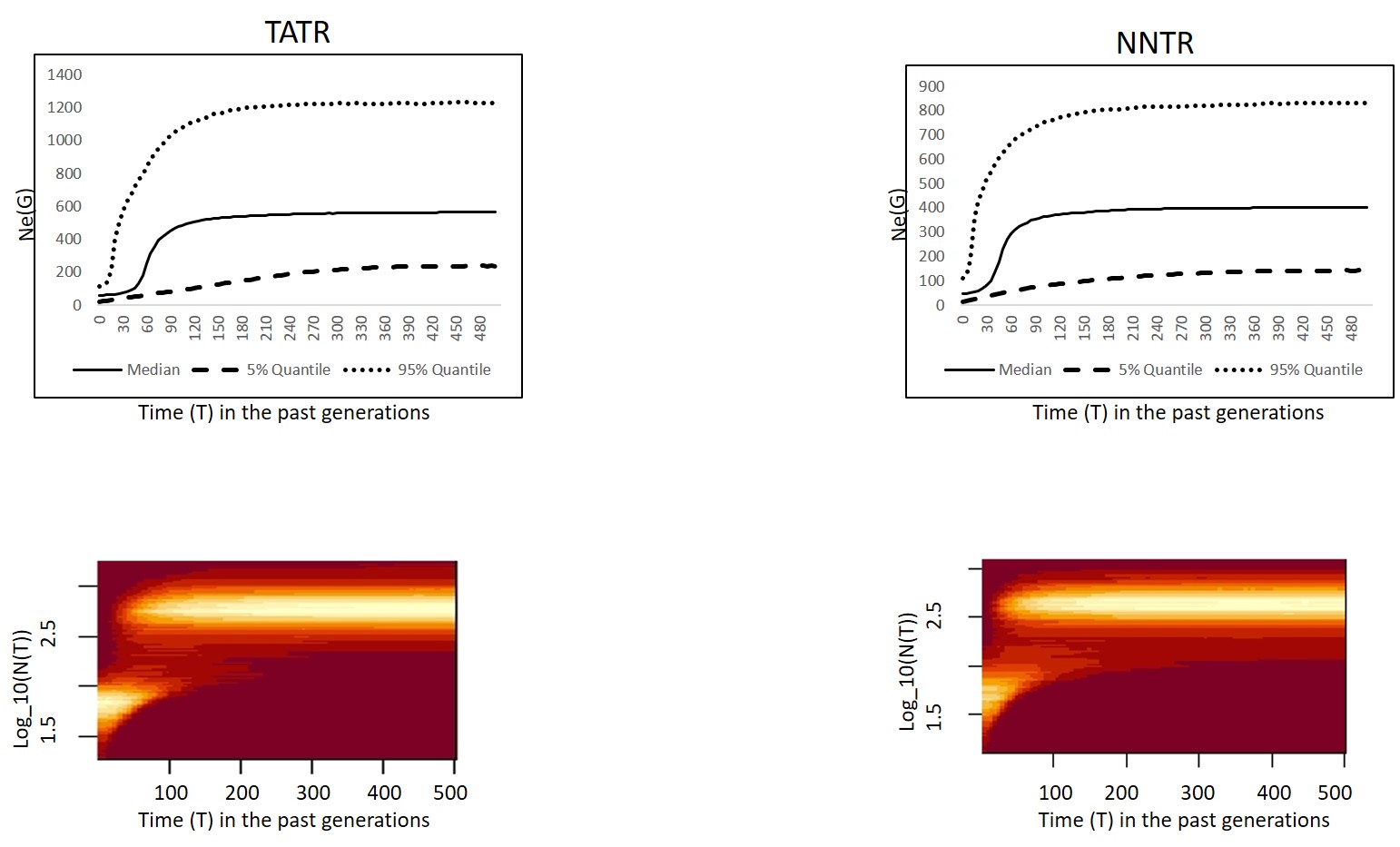


TATR

NNTR
